# Cell Therapy: A New Technology for Cerebral Circulation Restoration after Ischemia/Reperfusion

**DOI:** 10.32607/actanaturae.14338

**Published:** 2023

**Authors:** I. B. Sokolova, O. P. Gorshkova

**Affiliations:** Pavlov Institute of Physiology, Russian Academy of Sciences, St. Petersburg, 199034 Russian Federation

**Keywords:** ischemia/reperfusion, brain, intravenous transplantation, mesenchymal stem cells, microvascular density, reactivity, perfusion

## Abstract

Cell therapy with mesenchymal stem cells (MSCs) may be a promising technique for cerebral blood flow restoration after transient ischemia. Before a practical application of the cell material, 7–9 days are required for its cultivation. We studied the efficacy of human MSC (hMSC) transplantation performed 7 days after cerebral ischemia/reperfusion (I/R) to help recover cerebral circulation. The intravital micrograph technique was used to comparatively evaluate the vasculature density in the pia mater and the reactivity of the pial arteries in response to acetylcholine (ACh) in rats after I/R (clamping of both carotid arteries and a simultaneous decrease in and strict maintenance of the mean BP at 45 ± 2 mm Hg for 12 min) and with/without hMSC transplantation. Perfusion (P) in the sensorimotor cortex was assessed using laser dopplerography. After 14 and 21 days, the vasculature density in I/R-affected rats was 1.2- to 1.4-fold and 1.2- to 1.3-fold lower, respectively, than that in the controls. The number of ACh-dilated arteries decreased 1.6- to 1.9-fold and 1.2- to 1.7-fold 14 and 21 days after I/R, respectively. After 21 days, the P level decreased 1.6-fold, on average. Administration of hMSCs on day 7 after I/R resulted in complete recovery of the vasculature density by day 14. ACh-mediated dilatation fully recovered only in arteries of less than 40 μm in diameter within 21 days. After 21 days, the P level was 1.2-fold lower than that in the controls but significantly higher than that in rats after I/R without hMSCs. Delayed administration of MSCs after a transient cerebral ischemic attack affords the time for the procedures required to prepare cell material for transplantation and provides a good therapeutic response in the pial microvasculature.

## INTRODUCTION

Today the concept of a neurovascular unit (NVU) is widely used in the study of ischemic brain pathologies [1]. NVU is a structural and functional unit that comprises neurons, glial cells, astrocytes, pericytes, and vessels that provide gas and metabolic exchange [2]. NVU is involved in the regulation of the blood flow through the contractility of pericytes in the capillary bed [3] and smooth muscle cells (SMCs) in the arterial walls [4]. The key factor in NVU recovery after transient ischemia is the reactivity of NVU arteries [5]. Cell therapy using mesenchymal stem cells (MSCs) may be one of the most promising modern techniques to restore the structure and ability to function of the brain vasculature after transient ischemia [6]. However, practical application of cell material requires time to culture MSCs. If the patient’s MSCs are isolated in advance and stored in a cryobank, it would take 7–9 days to produce the required amount of cell material [7].

The aim of this study was to elucidate the effect of intravenous hMSC transplantation, which was performed 7 days after ischemia/reperfusion, on the vasculature density, pial artery reactivity, and tissue perfusion in the cerebral cortex 14 and 21 days after ischemia.

## MATERIALS AND METHODS

The study was performed in animals received from the Center for Collective Use “Biocollection of the Pavlov Institute of Physiology of the Russian Academy of Sciences for the Investigation of the Integrative Mechanisms of Nervous and Visceral System Activity” (Saint-Petersburg). The study was conducted in accordance with the regulations of the Ministry of Health and Social Development of the Russian Federation No. 708n of August 23, 2010 “Rules for Laboratory Practice”, Directive 2010/63/EU of the European Parliament and the Council of the European Union on the protection of animals used for scientific purposes, and the requirements of the Commission for Control over the maintenance and use of laboratory animals at the Pavlov Institute of Physiology of the Russian Academy of Sciences (protocol No. 09/05 of 05.09.2022).

**Animals**

The experiments were performed on male Wistar rats (*n *= 68). The animals were housed under standard vivarium conditions with natural light and free access to water and food.

**Ischemia/reperfusion**

Ischemia was induced in chloral hydrate-anesthetized rats (intraperitoneally, 43 mg/100 g of body weight) by 12-minute occlusion of both carotid arteries and simultaneous controlled hypotension (reduction and strict maintenance of blood pressure (BP) at 45 ± 2 mm Hg by drawing/reinfusion of blood into a heparinized syringe). BP was directly measured through a femoral artery catheter connected to a DTX PlusTM transducer (Argon Critical Care Systems, Singapore) attached to a computer running original BP monitoring software developed in our laboratory. After the ischemia period, the withdrawn blood was completely reinfused. After suturing the surgical wounds and anesthesia recovery (on heated tables), the animals were returned to their standard cages.

**MSCs and their transplantation**

hMSCs derived from a single donor were used for intravenous transplantation. MSC isolation from the bone marrow, culturing, and phenotyping were performed at Trans-Technologies LLC according to minimally modified standard procedures [8, 9]. In particular, hMSCs were cultured using α-MEM nutrient medium (Hyclone, New Zealand) supplemented with 20% fetal bovine serum (Gibco, USA) and 100 µg/mL penicillin/streptomycin (Gibco, USA). hMSCs were phenotyped using flow cytometry on a FACSscan flow cytometer (Becton Dickinson, USA). hMSCs were stained with anti-positive CD90, CD105, CD44, and CD73 and negative CD45, CD34, CD14, CD11b, HLA-DR, and 7AAD marker antibodies (Becton Dickinson, USA). MSCs at passage 2 or 3 were used for the transplantation. Intravenous transplantation was performed in separate groups of rats on day 7 after cerebral I/R. Each animal was injected with 5 million hMSCs in 30 μL of the culture medium.

All subsequent surgical and experimental procedures were performed on anesthetized (Zoletil, 20 mg/kg, ip, Virbac, France) rats; euthanasia was performed by administration of an increased Zoletil dose.

**Animal groups**

1. Control group: sham-operated (SO) Wistar rats that underwent surgery without I/R. The vasculature density, pial artery reactivity, and perfusion in the sensorimotor cortex in this and all subsequent groups in separate animal subgroups (acute experiments) were studied 14 and 21 days after surgery. Rat weight and BP were 303 ± 12.7 g and 133 ± 5 mm Hg, respectively, on day 14 (*n *= 10) and 330 ± 12.2 g and 135 ± 2 mm Hg, respectively, on day 21 (*n *= 9).

2. Wistar rats that underwent brain I/R. Weight and BP were 256 ± 5 g and 133 ± 5 mm Hg, respectively, on day 14 (*n *= 8) and 318 ± 4 g and 124 ± 4 mm Hg, respectively, on day 21 (*n *= 9).

3. Wistar rats that underwent brain I/R and were intravenously injected with hMSCs on day 7. Weight and BP were 340 ± 4.5 g and 128 ± 4 mm Hg, respectively, on day 14 (*n *= 10) and 336.7 ± 8.4 g and 132 ± 3.1 mm Hg, respectively, on day 21 (*n *= 10).

**Imaging and monitoring of the microvasculature**

A hole (S ≈ 1 cm^2^) was drilled in the parietal area of the animal’s skull to intravitally monitor pial artery response. The dura mater within the hole was removed, thereby opening the area for further study. The brain surface was continuously irrigated with a Krebs solution (pH 7.4) at 37°C. The mean BP was monitored and maintained at an approximately constant level throughout the experiment. The body temperature of the animals was maintained at 38°C throughout the experiment. Perfusion (P) in the cerebral cortex tissue was measured using a multifunctional laser diagnostic complex LAKK-M (LAZMA, Russia). A sensor of the device was placed at 3 points over the sensorimotor cortex with approximate coordinates AP = 1, 2, and 3 mm from the bregma; SD = 1.0 mm lateral to the sagittal suture. The LAKK-M complex software automatically calculated the mean microcirculation P.

The pial arteries were visualized in the same experimental animals using an original setup that included an MC-2ZOOM stereoscopic microscope (Micromed, Russia), a DCM-510 SCOPE digital camera for a microscope (Scopetek, China), and a personal computer. The number of arteries and the total number of vessels in a certain area were evaluated in static images using the PhotoM cytophotometry software (developed by A. Chernigovsky, http://www.t_lambda.chat.ru). The vasculature density was calculated as the vessel number to area ratio (units/μm2). Then, pial artery diameters were measured. During the experiment, 40 to 120 pial arteries were examined in each animal. The diameter of the arteries was measured under standard conditions under continuous irrigation of the brain surface with the Krebs solution and an acetylcholine (ACh) solution (10-7 M) (Sigma-Aldrich, USA). All examined pial arteries were divided into groups according to their diameters: 60–80 µm, 40–60 µm, 20–40 µm, and less than 20 µm. The effect of ACh was assessed based on the number of dilated arterial vessels and their degree of dilatation. A change in the number of ACh-dilated vessels was expressed as a percentage of the total number of examined vessels in a group. The degree of dilatation ΔD was assessed as the difference in diameters after (D2) and before (D1) exposure to ACh divided by the vessel diameter D1 before exposure, %:

ΔD = (D2 –D1)/D1 × 100.

If a diameter change was less than 5.0 ± 0.5%, it was considered as a lack of response. As we had previously found, this value was detected at rest in the lack of any exposure. Data for each group of vessels from different animals were averaged for a separate experimental group of rats and used for statistical comparisons.

**Statistical evaluation of data**

Mathematical data processing was performed using the Microsoft Excel 2003 statistical package and InStat 3.02 software (GraphPad Software Inc., USA). The data are presented as the arithmetic mean and its error. Testing of experimental data for normal distribution was carried out using the Kolmogorov– Smirnov test. The means of independent samples with a normal distribution were compared using the analysis of variance, followed by pairwise comparison of the groups using the Tukey’s test. If the sample distribution was not normal, the groups were compared using the Kruskal–Wallis test, followed by pairwise comparison using the Mann–Whitney U-test. Differences were considered significant at a confidence level of more than 95% (*p * < 0.05).

## RESULTS

Flow cytometry analysis revealed that the hMSC culture was comprised of 99.7% CD90+, CD73+, CD105+, and CD44+ cells (true MSCs); 0.3% of CD45+ and CD34+ cells (hematopoietic cells); and 0.5% of CD14+, CD11b+, and HLA-DR+ cells. The 7AAD+ (non-viable) cells accounted for less than 0.9–1%.

**Fig. 1:**
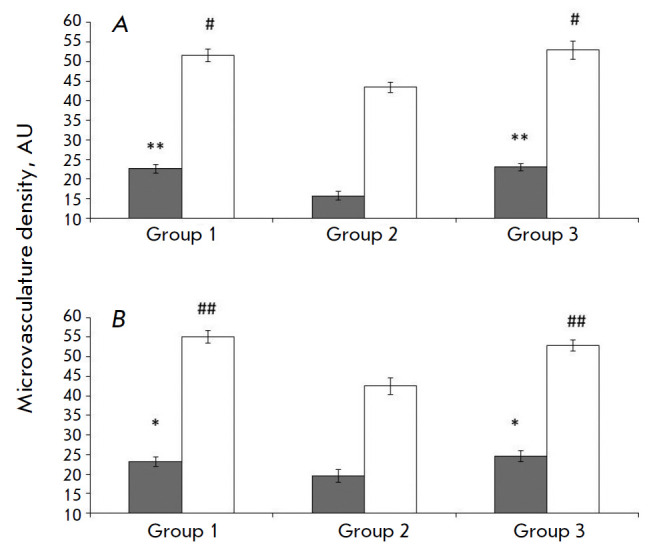
Indicator of the vascular bed density of the sensorimotor cortex pial membrane in the experimental animals. (*A*) – 14 days after I/R, (*B*) – 21 days after I/R. *Dark columns *– the artery density; *light columns *– the density of the all-vascular network.* Horisontally *– experimental animals groups; *vertically *– indicator of the microvascular bed density (number of vessels / unit area). * – changes in the arterial density are significant compared to the corresponding values in the animals after I/R; # – changes in the density of the all-vascular network are significant compared to the corresponding values in the animals after I/R (*, # *p* < 0.05, **, *## p* < 0.01, Tukey test)

The microvasculature density in the pia mater of the sensorimotor cortex in the SO and I/R rats is shown in *Fig. 1*. The microvasculature density and the arterial vessel density in the I/R group were lower, 1.4-fold and 1.2-fold, respectively, than those in the SO group 14 days after I/R and 1.2-fold and 1.3-fold, respectively, 21 days after I/R. The microvasculature density in the pia mater of I/R group animals subjected to intravenous hMSC transplantation was the same as that in the SO rats both on days 14 and 21 after I/R.

**Fig. 2:**
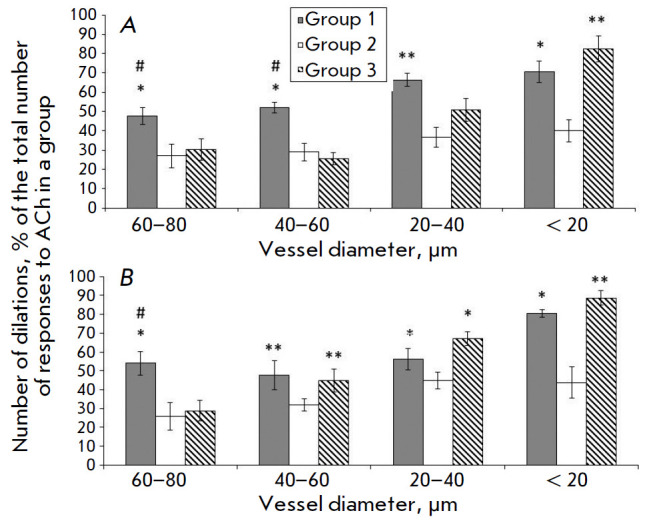
The number of pial arteries that responded with dilation to the effects of ACh. (*A*) – 14 days after I/R, (*B*) – 21 days after I/R. *Dark columns *– sham-operated rats, *light columns *– rats after I/R, *oblique hatching *– rats after I/R, with intravenous transplantation of MSCc 7 days after I/R. *Horizontally *– vessels diameter, *vertically* – number of vessels dilated in response to ACh, % of the total number of reactions to ACh in the group. * – significant changes in comparison with the corresponding values in the rats after I/R; # – significant changes in comparison with the corresponding values in the rats after I/R, with intravenous transplantation of MSCc 7 days after I/R (*,#*p * < 0.05, **p < 0.01, Tukey test)

In the I/R group (the animals not treated with cell therapy), application of Ach to the brain surface significantly deteriorated the pial artery reactivity (*Fig. 2*). In the rats from group 2, the number of Achdilated arterial vessels (an increase in the diameter) was 1.8-fold and 1.3- to 2.1-fold, on average, less than that in the SO rats after 14 and 21 days, respectively.

**Fig. 3:**
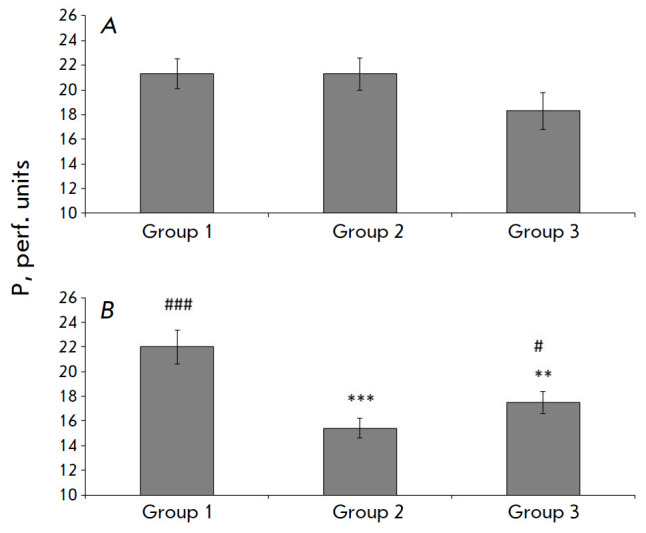
Changes in the perfusion index in the shamoperated and ischemic rats. (*A*) – 14 days after I/R, (*B*) – 21 days after I\R. *Horizontally *– groups of experimental animals; *vertically *– perfusion (perf. units). ** – significant changes in comparison with the corresponding values in the sham-operated rats of this group; # – significant changes in comparison with the corresponding values in the rats after I/R (#*p* < 0.05, **p < 0.01, ***, ###*p* < 0.001, Mann-Whitney U-test)

In the group of animals injected with hMSCs on day 7 after I/R, the dilatation response of large pial arteries (> 40 μm in diameter) was 1.3- to 2-fold, on average, lower than that in the SO rats; i.e., approximately the same as in the animals of the 2^md^ group 14 days after I/R (7 days after hMSC injection). The number of ACh-dilated arteries with a diameter of 20–40 µm was lower (1.3-fold, on average) than that in the SO rats and higher than that in group 2 (1.4-fold, on average). In the smallest arteries ( < 20 μm in diameter), the dilatation response to ACh fully recovered to the level of that in the SO rats. The reactivity of the largest arteries (60–80 µm in diameter) had not recovered by day 21 after I/R (14 days after hMSC injection). The number of ACh-dilated arteries with a diameter of 20–60 μm was almost identical to that in the SO rats; this indicator for the vessels with a diameter of less than 20 µm was statistically significantly higher than that in the SO group. There were no differences between the groups in the degree of diameter changes (data not shown).

After 14 days, the P level in the sensorimotor cortex tissue in all I/R animals was still approximately identical to that in the SO rats (*Fig. 3*). On day 21, a significant decrease in P (1.6-fold, on average) was observed in group 2. In the animals from the cell therapy groups, a decrease in P, but less significant (1.2-fold, on average), was also detected on day 21 after ischemia.

## DISCUSSION

The introduction of cellular technologies in medical practice requires that we develop techniques for the transplantation of MSCs that are quite remote from the site of the transient ischemia, ischemic stroke, brain injury, etc. However, treating these brain pathologies with MSCs requires accounting for the permeability of the blood–brain barrier for these cells. There is data in the literature indicating that there is an increase in the permeability of the blood–brain barrier during the first 7 days after I/R [10], which enables venously transplanted MSCs to migrate to the brain. MSCs, administered intravenously 24 h after occlusion of the middle cerebral artery, have been experimentally shown to migrate into the damaged brain tissue and appear in the walls of penumbra vessels [11]. Increased levels of the vascular endothelial growth factor (VEGF) and hypoxia-induced factor (HIF-1α) have been observed in the same area. MSCs secrete factors that promote tissue neovascularization: fibroblast growth factor 2 (FGF-2), VEGF, transforming growth factor (TGFβ), interleukins IL-6 and IL-8, angiogenin, hepatocyte growth factor (HGF), and platelet- derived growth factor (PDGF BB) [12]. In addition to angiogenesis activation, MSCs can protect cerebral vascular cells after an ischemic stroke [13, 14].

In this study, we were able to show that the vasculature density in the pia mater of rats transplanted with hMSCs on day 7 after I/R was approximately identical to that in the SO animals (*Fig. 1*) on days 14 and 21 after ischemia. I/R is known to be followed by the formation of ischemic areas in brain tissue [15, 16, 6]. Tissue ischemia stimulates the proliferation of hMSCs and enhances their paracrine function [17]. MSCs cultured under hypoxic conditions increase the production of the vascular endothelial growth factor (VEGF) and hypoxia-induced factor (HIF-1α) [18]. We contend that the recovery of the vasculature structure, following hMSC transplantation on day 7 after I/R in our experiments, occurred due to hMSC-activated angiogenesis. This insight is also confirmed by the ACh-induced reactivity of the pial arteries. We showed that ACh-mediated dilatation of large arteries (> 60 μm in diameter) did not recover after 14 or 21 days (*Fig. 2*). The dilatation response of arteries with a diameter of 20–40 µm was lower than that in the animals in the control group on day 14. Probably, damaged endothelial cells in the large vessels failed to recover due to the administration of hMSCs 7 days after I/R. In the smallest arteries ( < 20 µm in diameter), the reactivity was the same as that in the control group as early as on day 14 (i.e., 7 days after administration of hMSCs). On day 21, the number of ACh-induced dilatations was statistically greater than that in the control group and the group of cell therapy performed on the day of the I/R. This confirms the activation of angiogenesis in ischemic brain tissue after hMSC transplantation on day 7 after I/R. In this case, there was also a paracrine therapeutic effect on the vascular wall. This is seen in arteries with a diameter of 20–60 µm, in which reactivity had not recovered to its control level by day 14 after I/R and was identical to that in the SO group by day 21 (14 days after administration of hMSCs) (*Fig. 2*). Probably, the ability to function of the endothelial cells in these vessels was damaged by I/R, but the cells survived and were able to recover thanks to MSC-secreted trophic factors [19, 20]. For example, increased production of HIF-1α may be considered as a therapeutic effect after I/R. In ischemic tissue, HIF-1α stimulates enhanced expression of the genes that provide cell adaptation to hypoxia and regulate vascular tone, cell proliferation, and apoptosis [21].

Restoration of the structure and functionality of the vasculature after I/R is very important for maintaining the physiological level of a cerebral blood flow rate. After a brief ischemic attack, when the cerebral blood flow rate abruptly falls, reperfusion leads to hyperemia. After 7–14 days, the blood flow rate usually drops to its initial level, but not always. This requires normalization of the blood gas composition (pO_2_ and pCO_2_) and acid-base balance (pH), activation of secondary angiogenesis, and restoration of a balanced production of vasoconstrictors and vasodilators by endothelial cells [22]. However, in a real-world situation, there may be a decrease in the microvasculature density, endothelial dysfunction (as in this study), compression of the vessel lumen by swollen processes of astrocytes, and intravascular accumulation of erythrocytes, platelets, and leukocytes in the brain by day 21 after I/R [23]. These developments worsen cerebral circulation. The use of hMSCs on day 7 after I/R maintained tissue perfusion (an integral indicator of blood circulation) at a higher level than that in the non-treated rats (*Fig. 3*).

## CONCLUSION

We found that intravenous transplantation of hMSCs on day 7 after I/R led to good therapeutic results: the animals retained/recovered the vasculature structure in the pia mater. Delayed administration of MSCs 7 days after a transient ischemic attack buys time for the procedures required for the production of cell material for transplantation and completely restores arterial reactivity in the microcirculatory region of the pial vasculature.

## Abbreviations

BP: blood pressure
I/R: ischemia/reperfusion
SOR: sham-operated rat
MSCs: mesenchymal stem cells
hMSCs: human mesenchymal stem cells
P: perfusion
ED: endothelial dysfunction
ACh: acetylcholine
